# On-treatment mortality predictors in chronic hepatitis B patients experiencing severe acute exacerbation: a prospective observational study

**DOI:** 10.1186/1756-0500-6-349

**Published:** 2013-09-02

**Authors:** Yi-Cheng Chen, Chao-Wei Hsu, Ming-Yang Chang, Chau-Ting Yeh

**Affiliations:** 1 Department of Gastroenterology-Hepatology, Liver Research Unit, Division of Hepatology, Taipei, Taiwan; 2 Kidney Research Center, Chang Gung Memorial Hospital, Chang Gung University College of Medicine, Taipei, Taiwan; 3 Liver Research Center, Chang Gung Memorial Hospital, 199, Tung Hwa North Road, Taipei, Taiwan

**Keywords:** Severe acute exacerbation, Chronic hepatitis B, Alpha-fetoprotein, Estimated glomerular filtration rate

## Abstract

**Background:**

Severe acute exacerbation in chronic hepatitis B could lead to mortality in some patients unless timely liver transplantation is performed. The baseline bilirubin level has been reported to be an important prognostic factor for mortality. Here we conducted a prospective observational study to examine the clinical performance of this predictor.

**Method:**

Twenty-one consecutive chronic hepatitis B patients experiencing severe acute exacerbation were treated with either telbivudine or entecavir. The clinical characteristics at baseline and week-2 were documented and correlated with mortality.

**Results:**

Of the 21 patients included, 9 had baseline bilirubin >10 mg/dL. Four of these 9 patients (44.4%) eventually died, whereas all other patients survived. During the initial 2-week period, the change of bilirubin was −1.2 mg/dl in the survivors, but was +8.05 mg/dl in the mortalities (P = 0.009). When this on-treatment factor was combined, 5 of the 21 patients had baseline bilirubin > 10 mg/dL plus an increase of bilirubin level at week-2. Of these 5 patients, 4 (80%) died. Thus, by combining the baseline and on-treatment bilirubin levels, a positive predictive value of 80% and a negative predictive value of 100% could be achieved. Other significant on-treatment mortality predictors (at week-2) included higher international normalized ratio of prothrombin time (2.75 vs. 1.3, P = 0.004), higher model for end-stage liver disease score (30 vs. 17, P = 0.006), lower alpha-fetoprotein level (36.3 vs. 459.6 ng/mL, P = 0.039), and more rapid deterioration of the estimated glomerular filtration rate (eGFR) (P = 0.008). Interestingly, during the course, deterioration of eGFR was statistically significant in entecavir-treated (P = 0.028), but not in telbivudine-treated patients. Additionally, the patients treated with telbivudine had significant increase in serum alpha-fetoprotein (27.9 to 191.9 ng/ml, P = 0.046) in the first 2 weeks, whereas the corresponding feature was not found in those treated with entecavir (P = 0.139).

**Conclusions:**

In this prospective observational study, we discovered that the baseline and on-treatment bilirubin levels should be combined to achieve a better predictive value. Telbivudine might have a renoprotective effect in addition to its efficacy in viral suppression in patients with severe acute exacerbation.

## Background

Severe acute exacerbation (SAE) is not uncommon in the natural course of chronic hepatitis B virus (HBV) infection, characterized by high serum alanine aminotransferase (ALT) level, jaundice, coagulopathy and hepatic decompensation [1-4]. The prognosis is poor with a high mortality of 65-93% once hepatic encephalopathy has occurred [4-6]. Thus, timely liver transplantation is indicated. The prognostic factors for mortality include high serum bilirubin level, thrombocytopenia or liver cirrhosis, prolonged prothrombin time or high international normalized ratio (INR) of prothrombin time, low serum albumin level at baseline and emergence of ascites or encephalopathy during the course [3,7-9]. However, because of the retrospective nature of these studies, our knowledge regarding the clinical usefulness of on-treatment factors in predicting mortality is very limited. Furthermore, more and more potent antiviral drugs are now approved for clinical use. Conceivably, effective antiviral therapy would improve the survival of patients with SAE and thus change the predictivity of previously identified factors.

Lamivudine, a nucleoside analogue with inhibitory effects on HBV polymerase/reverse transcriptase activity, has been used as a rescue therapy for SAE in chronic hepatitis B patients. However, the mortality rate remains high, which varies from 13.5 to 38% in studies from different populations [3,7-11]. Entecavir (ETV) is a potent inhibitor of HBV DNA polymerase with a high resistance barrier and Telbivudine (LdT) is an orally bioavailable L-nucleoside with rapid HBV suppression activity. Both have been approved for treatment of chronic hepatitis B and have superior antiviral activity to lamivudine [12-15]. ETV has been used in decompensated chronic hepatitis B in clinical trials [16,17] as well as in clinical practices [18,19] with satisfactory safety profile and tolerability. On the other hand, the results from a 2-year randomized clinical trial in treatment of decompensated HBV-related cirrhosis also showed that LdT was well tolerated and the treatment led to stabilization of liver function [20]. Despite the availability of these evidences, the clinical data regarding LdT usage in chronic hepatitis B patients with SAE remain scanty.

Since all prognostic predictors for mortality are obtained from retrospective studies, it is unclear whether the performance of these predictors is satisfactory in real world, especially when patients with SAE are now being treated with potent antiviral drugs. Of the baseline prognostic predictors, bilirubin level has been repeatedly reported to be an important factor for mortality. In this prospective observational study, we treated the SAE patients with either ETV or LdT and examined the performance of the baseline bilirubin level together with other mortality predictors. Additionally, we explored the early on-treatment factors as accessory predictive factors for mortality.

## Methods

This was an open label, prospective observational study of patients with chronic hepatitis B, admitted for spontaneous SAE to Chang Gung Memorial Hospital since July 2009. The study was approved by the Institutional Review Board of Chang Gung Medical Center and written informed consent was obtained from all patients enrolled. The SAE of chronic hepatitis B was defined as elevation of ALT to greater than five times of upper limit of normal (ULN, 36 U/L) with serum total bilirubin level greater than 2 mg/dL and INR ≥ 1.3. The antiviral therapy with nucleoside analogue was given under the coverage of the national health insurance in Taiwan, because of the clinical status of hepatic decompensation. Patients with coinfection of hepatitis C virus, hepatitis D virus or human immunodeficiency virus and alcoholic liver disease were excluded.

Twenty-one consecutive patients fulfilling the definition of SAE, treated with LdT 600mg/day (n = 9) or ETV 0.5mg/day (n = 12), were included in this study. All antiviral treatments were initiated within 1 week of symptom onset. No antiviral treatment had been given prior to the onset of this SAE. The decision of antiviral drug was made by each patient and physician after discussion. The factors under consideration included risk of resistance, oncogenic potential in animal study, and possible renal function preservation. All patients were followed at baseline, weeks 2, 4, 8, 12 and then every 3 months for liver biochemistry, INR, alfa-fetoprotein (AFP), creatinine and estimated glomerular filtration rate (eGFR). Model for end-stage liver disease (MELD) was calculated as described in the literature [21] at the corresponding time points. The eGFR was calculated by modification of diet in renal disease (MDRD) formula [22] and Chronic Kidney Disease Epidemiology Collaboration (CKD-EPI) creatinine equation [23]. Serum HBV DNA was tested at baseline, weeks 2, 4, 12 and then every 3 months using a standardized automated quantitative polymerase chain reaction assay (Roche COBAS TaqMan HBV Test, Roche Diagnostics, Pleasanton, CA) with a detection range of 69 to 6.402 × 10^6^ copies/mL.

Statistical analyses were performed by Statistical Package for Social Science Statistics (SPSS Statistics, version 17.0, Chicago, IL, USA). Continuous variables were expressed as median (range) and were compared by Mann–Whitney U test. Categorical variables were compared by Chi-square test or Fisher’s exact test as appropriate. HBV DNA level was logarithmic transformed for analysis. The paired comparison of two relative variables was performed by Wilcoxon signed rank test. P<0.05 was considered statistically significant.

## Results

### The baseline and week-2 clinical parameters associated with mortality

The median (range) age was 48 (28–71) years and 18 (86%) were males. The median (range) follow-up period was 19.4 (0.8-28.2) months. All patients had a known history of chronic hepatitis B with positive serum HBV surface antigen (HBsAg) detected at least 1 year before SAE. All patients were negative for IgM class anti-HBV core antibody. Three patients had liver cirrhosis at the time of SAE and three had ascites due to liver failure. Four patients died at the time of 0.8, 1.0, 1.9 and 2.5 months during follow-up with 1-month and 3-month mortality rate of 9.5% and 19%, respectively. The overall mortality rate was 19% (2 with LdT, 22.2%; 2 with ETV, 16.7%).

The median levels of serum total bilirubin, INR and MELD scores at baseline in the mortalities were 20.6 mg/dL, 3.3 and 31.5, respectively. These were significantly higher than the corresponding figures in those who survived (7.7 mg/dL, P = 0.007; 1.5, P = 0.002; and 19, P = 0.003, respectively). The difference was also statistically significant in serum albumin (2.95 vs. 3.6 mg/dL, P = 0.020) and HBV DNA level (5.06 vs. 8.16 log_10_ copies/mL, P = 0.031) between patients with mortality and survival, as shown in Table 1. The serum ALT, creatinine, eGFR by MDRD and CKD-EPI, platelet count and AFP were not statistically different in the mortalities and survivals.

**Table 1:** Baseline clinical characteristics by different outcomes and treatment groups

	**All**	**Survival**	**Mortality**	**p**	**Telbivudine**	**Entecavir**	**p**
**Patient no**	**21**	**17**	**4**		**9**	**12**	
Age	48 (28–71)	49 (28–71)	36.5 (29–51)	0.226	48 (29–65)	47 (28–71)	0.859
Gender				1.000			1.000
Male	18	14	4		8	10	
Female	3	3	0		1	2	
LdT/ETV	9/12	7/10	2/2	1.000	−	−	−
Mortality	−	−	−	−	2	2	1.000
HBeAg+	9	7	2	1.000	3	6	0.660
Cirrhosis	3	2	1	0.489	1	2	1.000
Ascites	3	1	2	0.080	1	2	1.000
Encephalopathy	1	1	0	0.190	1	0	0.429
FU (M)	19.4 (0.8–28.2)	25.0 (4.0–28.2)	1.45 (0.8–2.5)	0.002	5.1 (1.9–28.2)	24.7 (0.8–27.7)	0.722
Albumin	3.49 (2.5–4.9)	3.6 (2.5–4.9)	2.95 (2.5–3.3)	0.020	3.6 (2.9–4.2)	3.4 (2.5–4.9)	0.477
ALT	1270 (146–2480)	1270 (211–2480)	1197 (146–2247)	0.929	1523 (211–2480)	870 (146–2247)	0.177
Bilirubin T	9.2 (2.1–33.1)	7.7 (2.1–16.8)	20.6 (11.9–33.1)	0.007	8.6 (2.1–20.4)	10.25 (2.2–33.1)	0.356
Creatinine	0.68 (0.39–1.46)	0.71 (0.47–1.34)	0.48 (0.39–1.46)	0.165	0.71 (0.47–1.05)	0.63 (0.39–1.46)	0.972
eGFR							
MDRD	116 (53–254)	116 (53–183)	194.5 (54–254)	0.106	116 (73–201)	127 (53–254)	0.831
CKD-EPI	111.1 (52.9–156.7)	110.1 (52.9–131.1)	138.2 (59.8–156.7)	0.152	112 (77.8–148)	110.6 (52.9–156.7)	0.722
Platelet (10^3^)	148 (44–253)	148 (62–253)	121.5 (44–174)	0.244	174 (44–251)	121.5 (62–253)	0.188
INR	1.6 (1.3–3.5)	1.5 (1.3–2.3)	3.3 (2.6–3.5)	0.002	1.5 (1.3–3.1)	1.6 (1.4–3.5)	0.773
AFP	65.7 (4.1–650.7)	49.4 (4.1–477.2)	109.5 (27.9–650.7)	0.325	27.9 (4.2–238.3)	162.1 (4.1–650.7)	0.088
HBV DNA (log_10_ cps/ml)	8.04 (4.3–9.68)	8.16 (5.22–9.68)	5.06 (4.30–8.20)	0.031	8.04 (4.97–9.47)	8.04 (4.30–9.68)	0.886
MELD score	20 (13–37)	19 (13–28)	31.5 (26–37)	0.003	19 (14–31)	20 (13–37)	0.412

Data shown as median (range); *eGFR* estimated glomerular filtration rate, *MDRD* modification of diet in renal disease (mL/min/1.73m^2^), *CKD-EPI* Chronic Kidney Disease Epidemiology Collaboration (mL/min/1.73m^2^), *INR* international normalized ratio, *AFP* alpha-fetoprotein, *MELD* model for end-stage liver disease.

At week 2, patients with mortality had significantly higher serum total bilirubin (29.1 vs. 4.8 mg/dL, P = 0.003), INR (2.75 vs. 1.3, P = 0.004) and MELD score (30 vs. 17, P = 0.006) than the corresponding figures of those who survived. On the contrary, the survival patients had significantly higher AFP (459.6 vs. 36.3 ng/mL, P = 0.039), as shown in Table 2. There was no significant difference in serum ALT, creatinine, eGFR (MDRD and CKD-EPI) and HBV DNA levels at week 2 between the survivals and the mortalities.

**Table 2:** Paired comparison of clinical parameters between baseline and week 2 in different outcomes and treatment groups

	**Overall**			**Survival**			**Mortality**			**Telbivudine**			**Entecavir**		
**Patient no**	**21**			**17**			**4**			**9**			**12**		
**Time point**	**Baseline**	**Week 2**	**p**	**Baseline**	**Week 2**^**a**^	**p**	**Baseline**	**Week 2**^**a**^	**P**	**Baseline**	**Week 2**^**b**^	**p**	**Baseline**	**Week 2**^**b**^	**p**
ALT	1270 (146–2480)	167 (41–485)	<0.001	1270 (211–2480)	184 (41–485)	<0.001	1197 (146–2247)	112.5 (66–190)	0.068	1523 (211–2480)	184 (80–435)	0.011	870 (146–2247)	133.5 (41–485)	0.002
Bilirubin T	9.2 (2.1–33.1)	6.8 (1.8–43.2)	0.728	7.7 (2.1–16.8)	4.8 (1.8–21.3)	0.309	20.6 (11.9–33.1)	29.1 (17.8–43.2)	0.068	8.6 (2.1–20.4)	8.0 (1.9–25.3)	0.515	10.25 (2.2–33.1)	5.15 (1.8–43.2)	1.000
Creatinine	0.68 (0.39–1.46)	0.94 (0.37–3.79)	0.002	0.71 (0.47–1.34)	1.0 (0.37–1.81)	0.019	0.48 (0.39–1.46)	0.92 (0.72–3.79)	0.068	0.71 (0.47–1.05)	0.90 (0.59–1.49)	0.051	0.63 (0.39–1.46)	1.03 (0.37–3.79)	0.021
eGFR															
MDRD	116 (53–254)	89 (18–241)	0.005	116 (53–183)	77 (37–241)	0.042	194.5 (54–254)	90.5 (18–129)	0.068	116 (73–201)	89 (37–147)	0.044	127 (53–254)	79 (18–241)	0.050
CKD-EPI	111.1 (52.9–156.7)	98.3 (18.9–134.5)	0.003	110.1 (52.9–131.1)	82.9 (36.8–134.5)	0.028	138.2 (59.8–156.7)	101.8 (18.9–126.4)	0.068	112 (77.8–148)	98.3 (40.9–126.4)	0.066	110.6 (5.29–56.7)	86.9 (18.9–134.5)	0.028
INR	1.6 (1.3–3.5)	1.4 (1.0–4.2)	0.066	1.5 (1.3–2.3)	1.3 (1.0–2.8)	0.062	3.3 (2.6–3.5)	2.75 (2.2–4.2)	0.577	1.5 (1.3–3.1)	1.7 (1.1–2.8)	0.440	1.6 (1.4–3.5)	1.35 (1.0–4.2)	0.058
AFP	65.7 (4.1–650.7)	305.5 (6.2–2802)	0.013	49.4 (4.1–477.2)	459.6 (35.4–2802)	0.005	109.5 (27.9–650.7)	36.3 (6.2–66.3)	0.180	27.9 (4.2–238.3)	191.9 (66.3–1462.5)	0.046	162.1 (4.1–650.7)	459.6 (6.2–2802)	0.139
HBV DNA (log_10_ cps/ml)	8.04 (4.3–9.68)	4.19 (3.07–5.89)	<0.001	8.16 (5.22–5.06)	4.2 (3.07–5.89)	0.001	5.06 (4.3–8.2)	3.64 (3.31–3.96)	0.180	8.04 (4.97–9.47)	3.96 (3.07–5.10)	0.018	8.04 (4.30–9.68)	4.20 (3.13–5.89)	0.005
MELD score	20 (13–37)	19 (11–49)	0.283	19 (13–28)	17 (11–32)	0.166	31.5 (26–.7)	30 (26–49)	0.655	19 (14–31)	20 (11–32)	0.674	20 (13–37)	18 (11–49)	0.469

Data shown as median (range); *eGFR* estimated glomerular filtration rate, *MDRD* modification of diet in renal disease (mL/min/1.73m^2^), CKD-*EPI* Chronic Kidney Disease Epidemiology Collaboration (mL/min/1.73m^2^), *INR* international normalized ratio, *AFP* alpha-fetoprotein, *MELD* model for end-stage liver disease.

^a^: comparison of week 2 data between survival and mortality, p values in order were 0.324, 0.003, 0.720, 0.788, 1.000, 0.004, 0.039, 0.160, 0.006.

^b^: comparison of week 2 data between Telbivudine and Entecavir, p values in order were 0.394, 0.776, 0.776, 0.803, 0.670, 0.642, 1.000, 0.242, 0.915.

### Changes of clinical parameters between baseline and week-2 for all patients

To understand the progressive changes of clinical parameters during the treatment course, comparison of clinical factors at baseline and week 2 for all patients was performed (Table 2). The serum ALT and HBV DNA decreased significantly (1270 to 167 U/L, P < 0.001 and 8.04 to 4.19 log_10_ copies/mL, P < 0.001, respectively) in the first 2-week of antiviral treatment with paired comparison (Table 2). The serum creatinine increased significantly from baseline to week 2 (0.68 to 0.94 mg/dL, P = 0.002) despite of treatment. Correspondingly, the eGFR calculated by MDRD (116 to 89 mL/min/1.73m^2^, P = 0.005) and CKD-EPI (111.1 to 98.3 mL/min/1.73m^2^, P = 0.003) decreased significantly during the 2-week period. The AFP increased significantly (65.7 to 305.5 ng/mL, P = 0.013) from baseline to week 2. There was no significant difference between baseline and week 2 in serum total bilirubin, INR and MELD score.

Similar progressive changes for all the clinical parameters were found in the survival group, including the declines in serum ALT and HBV DNA levels (1270 to 184 U/L, P < 0.001 and 8.16 to 4.2 log_10_ copies/mL, P = 0.001, respectively), the increases of serum creatinine (0.71 to 1.0 mg/dL, P = 0.019) and decreases of eGFR (MDRD, 113.5 to 76 mL/min/1.73m^2^, P = 0.042; CKD-EPI, 110.1 to 82.9 mL/min/1.73m^2^, P = 0.028), and the increases of AFP (49.4 to 459.6 ng/mL, P = 0.005) (Table 2).

### The changes (∆) of parameters between baseline and the week 2 time point in association with mortality

**Figure 1:**
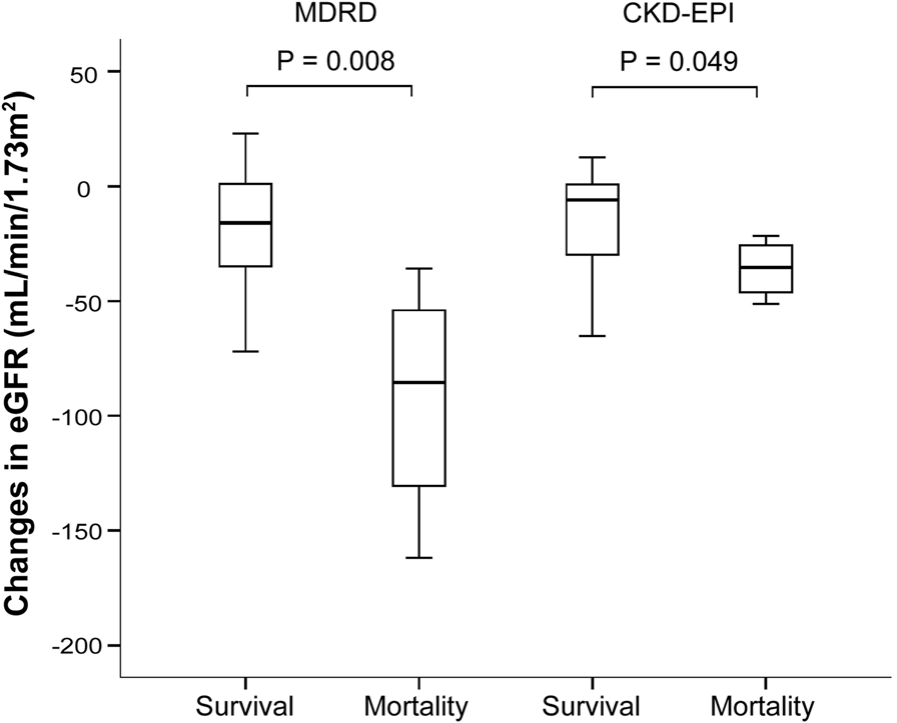
**The comparison of changes (∆) from baseline to week 2 between the patients with survival and mortality in estimated glomerular filtration rate.** Modification of diet in renal disease (MDRD), p=0.008 and Chronic Kidney Disease Epidemiology Collaboration (CKD-EPI), p=0.049. The boxplots showed the extreme values, 25th, 50th (median) and 75th percentiles and outliers.

When comparing the parameters in terms of the changes (∆) from baseline to week 2, the mortalities had significantly greater increase in serum total bilirubin (∆=+8.05 vs −1.2 mg/dL, P = 0.009) and creatinine (∆=0.49 vs 0.08 mg/dL, P = 0.049) levels, and more rapid decrease in eGFR (MDRD, ∆=−85.5 vs −16 mL/min/1.73m^2^, P = 0.008; CKD-EPI, ∆=−35.5 vs −6.0 mL/min/1.73m^2^, P = 0.049) (Figure 1). The serum AFP increased in the survivals (∆=184.2 ng/ml) but decreased in the mortalities (∆=−73.2 ng/ml) with marginal significance (P = 0.057). The survivals had HBV DNA decline of 3.4 log IU/mL and only 1.4 log IU/mL in the mortalities (P = 0.074) (Table 3).

**Table 3:** The changes (∆) of clinical parameters from baseline to week 2 in different outcomes and treatment groups

	**All**	**Survival**	**Mortality**	**p**	**Telbivudine**	**Entecavir**	**p**
**Patient no**	**21**	**17**	**4**		**9**	**12**	
ALT	−1161 (−2313-+106)	−1161 (−2313-+106)	−890 (−2057- -44)	1.000	−1383 (−2313-+106)	−547.5 (−2057- -30)	0.256
Bilirubin T	−0.4 (−9.1-+12.2)	−1.2 (−9.1-+11.5)	8.05 (5.9-12.2)	0.009	−0.9 (−6-+11.5)	−0.3 (−9.1-+12.2)	0.722
Creatinine	0.22 (−0.1-+2.33)	0.08 (−0.10-+0.87)	0.49 (0.23-2.33)	0.049	0.22 (−0.09-+0.87)	0.27 (−0.1-+2.33)	0.594
eGFR							
MDRD	−23 (−162-+58)	−16 (−72-+58)	−85.5 (−162- -36)	0.008	−23 (−99-+23)	−23 (−162-+58)	0.749
CKD-EPI	−16.1 (−65.2-+12.6)	−6.0 (−65.2-+12.6)	−35.5 (−51.3- -21.7)	0.049	−21.1 (−65.2-+6.81)	−13.0 (−51.3-+12.6)	0.776
INR	−0.3 (−1.0-+0.9)	−0.3 (−1.0-+0.9)	−0.4 (−0.5-+0.7)	0.318	−0.3 (−0.6-+0.9)	−0.3 (−1.0-+0.7)	0.971
AFP	151.5 (−140.7-+2326.6)	184.2 (−110.2-+2326.6)	−73.2 (−140.7- -5.8)	0.057	184.2 (−5.8-+1385.9)	101.2 (−140.7-+2326.6)	0.386
HBV DNA (log_10_ cps/ml)	−3.27 (−5.47- -0.55)	−3.4 (−5.5- -0.6)	−1.4 (−1.8- -1.0)	0.074	−3.4 (−5.0- -1.0)	−3.0 (−5.5- -0.6)	0.922
MELD score	−1.0 (−11-+14)	−1.0 (−11-+14)	0.0 (−1-+12)	0.344	−1.0 (−6-+14)	−0.5 (−11-+12)	0.775

Data shown as median (range); *eGFR* estimated glomerular filtration rate, *MDRD* modification of diet in renal disease (mL/min/1.73m^2^), *CKD-EPI* Chronic Kidney Disease Epidemiology Collaboration (mL/min/1.73m^2^), *INR* international normalized ratio, *AFP* alpha-fetoprotein, *MELD* model for end-stage liver disease.

Of the 17 survivors, 5 had bilirubin >10 mg/dL at baseline, a feature predicting high mortality according to previous studies [7-9]. As such, only 4 of 9 (44.4%) with baseline bilirubin level > 10 mg/dL resulted in mortality. The positive predictive value (PPV) using only baseline bilirubin level was 44.4%.

**Figure 2:**
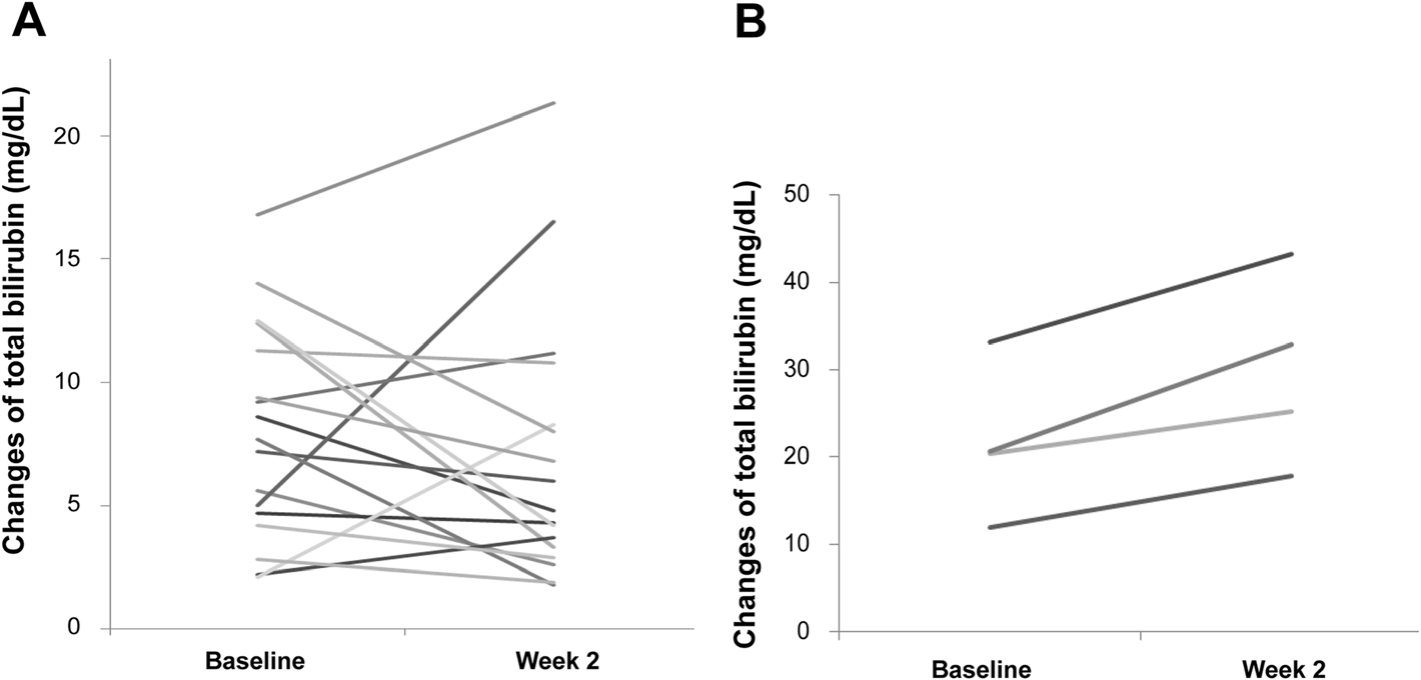
The graphical presentations of total bilirubin levels from baseline to week 2 in (A) 17 survivors and (B) 4 mortalities.

However, of these 9 patients, 4 of them showed decrease of bilirubin levels at week 2 and all these 4 patients survived (Figure 2). By using baseline bilirubin >10 mg/dL, combined with increase of bilirubin levels during the first 2-week period, 5 patients meeting these two criteria were identified. Four of these 5 patients resulted in mortality. The positive predictive value (PPV) was 80% and the negative predictive value (NPV) was 100% by use of the combination criteria.

### The efficacy of viral suppression and change of renal function in LdT and ETV treated patients

Of these patients, 9 were treated with LdT and 12 received ETV immediately after the diagnosis of chronic B hepatitis with SAE. As shown in Table 1, the baseline clinical characteristics were similar in patients receiving LdT and ETV. After 2 weeks of treatment, there was no statistical difference in the clinical parameters between the two treatment groups (Table 2). Two deaths occurred in each group (by 1.9, 2.5 months in LdT group and 0.8, 1.0 months in ETV group) during follow-up without significant difference in mortality rate (P = 1.000) (Table 1). Both LdT and ETV showed excellent effects on declines in serum ALT (1523 to 184 U/L, P = 0.011 and 870 to 133.5 U/L, P = 0.002, respectively) and HBV DNA levels (8.04 to 3.96 log_10_ copies/mL, P = 0.018 and 8.04 to 4.20 log_10_ copies/mL, P = 0.005, respectively) in the initial 2-week period of treatment. The patients treated with LdT had significant increase in serum AFP (27.9 to 191.9 ng/ml, P = 0.046) in 2 weeks, whereas the corresponding feature was not found in those with ETV treatment (P = 0.139).

Although serum creatinine levels at week 2 were higher than those at baseline in both LdT and ETV groups, the difference was statistically significant in ETV group (0.63 to 1.03 mg/dL, P = 0.021), but not in LdT group (0.71 to 0.9 mg/dL, P = 0.051). The decrease of eGFR using MDRD formula was significant in the LdT group (116 to 89 mL/min/1.73m^2^, P = 0.044) but not the ETV group (127 to 79 mL/min/1.73m^2^, P = 0.050). The decrease of eGFR using CKD-EPI equation was also statistically significant in ETV group (110.6 to 86.9 mL/min/1.73m^2^, P = 0.028), but not in LdT group (112 to 98.3 mL/min/1.73m^2^, P = 0.066). The changes (∆) from baseline to week 2 in serum ALT, total bilirubin, creatinine, eGFR (MDRD and CKD-EPI), INR, AFP, HBV DNA level and MELD score were similar in LdT and ETV groups without statistically significance (Table 3).

Until the last visits, all survived patients were uneventful during follow-up and no drug-resistance was found in patients treated with LdT.

## Discussion

This prospective observational study revealed not only the baseline but also the on-treatment predictors for mortality of chronic hepatitis B patients with hepatic decompensation secondary to SAE. In consistent with previous studies [3,7-9], the patients with mortality had significantly lower serum albumin, higher total bilirubin level and INR at baseline. The present study also showed significantly higher baseline MELD score in patients with mortality. At the week 2 time point, the serum total bilirubin, INR and MELD scores were significantly lower and AFP was significantly higher in the survivors (Table 2). The present results indicated that decreasing serum total bilirubin, INR and MELD score and increasing serum AFP at week 2 were good indicators for survival in SAE of chronic hepatitis B. In the meanwhile, it was noted that an increase in serum total bilirubin (baseline to week-2) in the patients combined with baseline bilirubin >10 mg/dl had the PPV of 80% for mortality, while the NPV was 100% for decreasing serum total bilirubin in the 2-week period. The overall mortality rate was 19% which was higher than that of a recent study in Hong Kong [19], but was lower when compared with the studies using lamivudine or tenofovir treatment [3,7,8,24]. The different study populations and timing to begin antiviral therapy may lead to this discrepancy.

Previous studies have shown that the HBV DNA level at the time of SAE had little correlation with short-term mortality [3,7-9,19]. However, the baseline HBV DNA level was significantly lower in patients with mortality in the present study. It might be attributive to the enhanced immune response in SAE, which led to massive hepatocellular necrosis and clearance of the majority of HBV. The results of aggravated liver injury and decreased liver reservation could increase the chance of mortality. At week 2, the HBV DNA levels were comparable between patients with survival and mortality, but the decline from baseline to week 2 was marginally greater in the survival patients (Table 3). Both LdT and ETV had excellent efficacy in viral suppression to high but not to low viral load at baseline. The virological decline was around 3 log_10_ IU/mL during 2-week period of LdT and ETV treatment. The rate of viral suppression was faster when compared with previous studies [12-15].

Serum AFP has been a marker of bridging hepatic necrosis in acute exacerbation of chronic hepatitis B when it is greater than 100 ng/mL [25], and an increasing level suggests the occurrence of hepatic regeneration in acute hepatitis or after partial hepatectomy [26,27]. Previous studies showed that decreasing serum AFP levels predicted poor prognosis of acute hepatic failure in patients with chronic hepatitis B and an increase in AFP level was strongly associated with a favorable outcome in patients with acetaminophen-induced liver injury [28,29]. The data in present study revealed that serum AFP levels in survival patients at week 2 were significantly greater than those in patients with mortality. It therefore could be inferred that the patients with increasing AFP levels likely had good hepatic regeneration and reservation and were able to survive in the SAE of chronic hepatitis B. However, the reason for significantly greater increase in AFP from baseline to week 2 in patients treated with LdT was unknown. But this could suggest that liver regeneration might be better under LdT treatment.

The deterioration of renal function is a prognostic factor indicating poor prognosis in liver disease [30]. In the present study, the serum creatinine was significantly higher at week 2 than at baseline with correspondingly significant decrease in eGFR by MDRD and CKD-EPI in all patients (Table 2). It meant that renal dysfunction did occur at the early stage of liver decompensation resulting from SAE. In view of the change (∆) from baseline to week 2, the deterioration of renal function was greater in those with mortality (Table 3). These results indicated that follow-up of serum creatinine with accompanied eGFR calculation is important in acute decompensated hepatitis patients for prediction of short term mortality and early preparation for liver transplantation. However, the serum creatinine production could decrease secondary to decreased hepatic creatine synthesis in severe liver disease and the eGFR can be overestimated [31]. Thus, the serum creatinine and eGFR in decompensated liver disease should be interpreted cautiously.

Since tenofovir was not available until 2011 June in Taiwan and the renal function was concerned, two nucleoside analogues, LdT and ETV, were used in present study. Both drugs are superior to lamivudine in viral suppression and treatment efficacy [12-15] and both had good safety profiles and tolerability in decompensated liver disease [16-20]. Of note was that the serum creatinine was significantly higher at week 2 than at baseline in ETV group, but not in LdT group. The same observation was noted in eGFR by CKD-EPI with statistically significant decrease in ETV group but not in LdT group. This observation correlated with the results of a recent clinical study of LdT in decompensated liver cirrhosis that the eGFR was significantly increased in LdT group [20]. Although the reason is still unknown, the nucleoside analogue of LdT seemed to be able to slow down or prevent the progression of renal impairment in acute decompensated hepatitis. This might be critical for these patients to gain more time for hepatic recovery and thus increase the chance of survival.

One of the limitations of this study is a small number of cases. This is partly due to the prospective nature of this observational study for validation purpose. Most chronic hepatitis B patients are now under antiviral treatment in Taiwan and thus the incidence of SAE is decreasing. In the future, if more patients can be included, a better clinical correlation can be made.

## Conclusions

In conclusion, increasing serum total bilirubin and deterioration of renal function were important on-treatment predictors of mortality in SAE-related decompensated liver disease. Increasing AFP might indicate good hepatic regeneration and thus constitute a favorable factor for survival. LdT treatment might preserve the renal function in addition to its efficacy in viral suppression.

## Abbreviations

SAE: Severe acute exacerbation; HBV: Hepatitis B virus; ALT: Alanine aminotransferase; INR: International normalized ratio; ETV: Entecavir; LdT: Telbivudine; AFP: Alpha-fetoprotein; eGFR: Estimated glomerular filtration rate; MELD: Model for end-stage liver disease; MDRD: Modification of diet in renal disease (MDRD); CKD-EPI: Chronic kidney disease epidemiology collaboration.

## Competing interests

This study was partly supported by a grant from Novartis, Taiwan. Otherwise, the authors have no financial and personal relationships with other people or organizations that could inappropriately influence (bias) their work.

## Authors’ contributions

YCC: patient recruitment, acquisition of data, analysis and interpretation of data, statistical analysis, drafting of the manuscript. CWH: patient recruitment, acquisition of data. MYC: analysis and interpretation of data. CTY: study concept and design, critical revision of the manuscript for important intellectual content, material support, study supervision. All authors read and approved the final manuscript.
